# *Carnitine palmitoyltransferase 1A* (*CPT1A*): a transcriptional target of PAX3-FKHR and mediates PAX3-FKHR–dependent motility in alveolar rhabdomyosarcoma cells

**DOI:** 10.1186/1471-2407-12-154

**Published:** 2012-04-25

**Authors:** Lingling Liu, Yong-Dong Wang, Jing Wu, Jimmy Cui, Taosheng Chen

**Affiliations:** 1 Department of Chemical Biology and Therapeutics, St. Jude Children's Research Hospital, 262 Danny Thomas Place, Memphis, TN, 38105, USA; 2 Hartwell Center for Bioinformatics and Biotechnology, St. Jude Children's Research Hospital, 262 Danny Thomas Place, Memphis, TN, 38105, USA

## Abstract

**Background:**

Alveolar rhabdomyosarcoma (ARMS) has a high propensity to metastasize, leading to its aggressiveness and a poor survival rate among those with the disease. More than 80% of aggressive ARMSs harbor a PAX3-FKHR fusion transcription factor, which regulates cell migration and promotes metastasis, most likely by regulating the fusion protein’s transcriptional targets. Therefore, identifying druggable transcription targets of PAX3-FKHR that are also downstream effectors of PAX3-FKHR–mediated cell migration and metastasis may lead to novel therapeutic approaches for treating ARMS.

**Methods:**

To identify genes whose expression is directly affected by the level of PAX3-FKHR in an ARMS cellular-context, we first developed an ARMS cell line in which PAX3-FKHR is stably down-regulated, and showed that stably downregulating PAX3-FKHR in ARMS cells significantly decreased the cells’ motility. We used microarray analysis to identify genes whose expression level decreased when PAX3-FKHR was downregulated. We used mutational analysis, promoter reporter assays, and electrophoretic mobility shift assays to determine whether PAX3-FKHR binds to the promoter region of the target gene. We used siRNA and pharmacologic inhibitor to downregulate the target gene of PAX3-FKHR and investigated the effect of such downregulation on cell motility.

**Results:**

We found that when PAX3-FKHR was downregulated, the expression of *carnitine palmitoyltransferase 1A* (*CPT1A*) decreased. We showed that PAX3-FKHR binds to a paired-domain binding-site in the *CPT1A* promoter region, indicating that *CPT1A* is a novel transcriptional target of PAX3-FKHR. Furthermore, downregulating *CPT1A* decreased cell motility in ARMS cells, indicating that *CPT1A* is a downstream effector of PAX3-FKHR–mediated cell migration and metastasis.

**Conclusions:**

Taken together, we have identified *CPT1A* as a novel transcriptional target of PAX3-FKHR and revealed the novel function of CPT1A in promoting cell motility. CPT1A may represent a novel therapeutic target for the treatment of ARMS.

## Background

Rhabdomyosarcoma (RMS) is the most common soft tissue sarcoma in children. Two subtypes of RMS have been identified on the basis of histopathologic features—embryonal (ERMS) and alveolar (ARMS)—each with distinct clinical and genetic characteristics. Most of the more aggressive ARMSs are associated with either a 2;13 or a 1;13 chromosomal translocation, generating PAX3-FKHR and PAX7-FKHR fusion products, respectively. The unique expression, function, and subcellular location of the fusion proteins contribute to their oncogenic behavior by modifying cell growth, differentiation, and migration [1].

ARMS has a high propensity to metastasize. Preventing metastasis is an important therapeutic approach to cancer treatment, and evidence shows that PAX3-FKHR may regulate cell migration, thus promoting a metastatic phenotype. Specifically, downregulating *PAX3-FKHR* in ARMS cells decreases cell migration and cell invasion [2]. In a preclinical mouse model of ARMS, the expression level of PAX-FKHR was low in preneoplastic skeletal muscle, but was >100-fold higher in ARMS tumors. Metastatic ARMS tumors expressed PAX3-FKHR at incrementally higher levels than the primary tumors, further demonstrating the roles of PAX3-FKHR in promoting tumor metastasis [3]. Although it is possible to prevent ARMS metastasis by downregulating PAX3-FKHR, transcription factors are challenging drug targets, and currently there is no pharmacologic inhibitor of PAX3-FKHR available. Therefore, identifying druggable transcription targets of PAX3-FKHR that are also downstream effectors of PAX3-FKHR–mediated cell migration and metastasis may lead to novel therapeutic approaches for treating ARMS.

Significant effort has been made to identify transcription targets of PAX3-FKHR, and several transcription targets of PAX3-FKHR that are involved in ARMS cell migration have been reported [4,5]. Although these studies have led to the identification of genes whose expression appears to be regulated by PAX3-FKHR in each individual study, very few genes have been identified in multiple studies, possibly due to the model systems used. In the present study, we use an ARMS model to identify genes whose expression is directly affected by the level of PAX3-FKHR in an ARMS cellular-context under physiologically relevant conditions. We have identified *carnitine palmitoyltransferase 1A* (*CPT1A*) as a novel PAX3-FKHR transcription target that also mediates the function of PAX3-FKHR in regulating cell motility.

CPT1 is anchored in the outer membrane of mitochondria and catalyzes the formation of long chain acyl-carnitine, which will then traverse the inner mitochondrial membrane and undergo β-oxidation in the mitochondria. Three isoforms of CPT1 have been identified: CPT1A, which is detected in many tissues and the most abundant in the liver; CPT1B, which is predominantly expressed in muscle; and CPT1C, which is mainly present in the brain. Knockouts of CPT1A and CPT1B are lethal [6,7], therefore the exact role of CPT1A or CPT1B in energy homoeostasis remains unresolved. CPT1C is not an essential gene in mice although the animals exhibit reduced fatty acid oxidation when CPT1C is knocked-out [8,9]. Although the precise role of CPT1 in tumor growth remains unknown, a recent study showed that CPT1C is frequently expressed in tumors and up-regulated in response to metabolic stress. Furthermore, CPT1C depletion reduced tumor growth in xenograft models [10]. Currently, how the expression of CPT1 is regulated in ARMS, and whether CPT1 plays a role in ARMS tumorigenesis or contributes to the aggressive metastatic behavior of ARMS have not yet been investigated. In this study, we establish that *CPT1A* is a transcription target of PAX3-FKHR. In addition, for the first time, we report that CPT1A regulates cell motility in ARMS cancer cells. Therefore, CPT1A is a transcription target of PAX3-FKHR and a downstream effector of PAX3-FKHR–mediated cell migration and metastasis, and may represent a therapeutic target for ARMS. Defining the regulation of CPT1A by PAX3-FKHR may facilitate the validation of CPT1A as a therapeutic target for treating ARMS.

## Methods

### Cell culture

Rh30, Rh41, RD, HEK293T, and NIH3T3 cells have been described previously [11,12]. All cells were cultured in an incubator with a humidified atmosphere maintained at 5% CO_2_ and 95% air at 37°C. Cells were split every 3 days at 90% to 95% confluency. Phenol red–free DMEM (Invitrogen, Carlsbad, CA) was used for all luminescence assays.

### Establishment of PAX3-FKHR–knockdown stable clones

Kikuchi et al [2] identified specific target sequence of PAX3-FKHR (GCCTCTCACCTCAGAATTC) and designed corresponding siRNA (GCCUCUCACCUCAGAAUUC) to specifically target PAX3-FKHR without affecting either PAX3 or FKHR. A control siRNA (CUACUAUACCGAUACUCCC) was used as a non-targeting control in their studies [2]. In order to stably knock-down PAX3-FKHR, identical DNA sequence (GCCTCTCACCTCAGAATTC for PAX3-FKHR; CTACTATACCGATACTCCC for control) was synthesized and subcloned into the pSUPER.retro.puro vector (OligoEngine, Seattle, Washington) according to the manufacturer’s protocol. The resulting constructs were transfected into Phoenix packaging cells (Orbigen, San Diego, CA) by using FuGENE 6 (Invitrogen). Viral particles were collected 72 hours after transfection and transduced into Rh30 cells. Puromycin (Invitrogen; 1 μg/mL) was used to select stable clones for both non-targeting control (CON) and PAX3-FKHR knock-down (KD). KD1 and KD2 are two stable clones from the same vector-based siRNA targeting PAX3-FKHR, and have the same growth rate ( Additional file 1: Figure S1).

### Real-time reverse transcription– polymerase chain reaction (RT-PCR)

Real-time RT-PCR was used to measure the levels of mRNA. Extraction of total RNA from cellular lysates, preparation of cDNA, the PAX3-FKHR and glucose-6-phosphate dehydrogenase (GAPDH) primers for PCR, and the conditions for PCR have been described previously [11]. *CPT1A* sense primer: TCCAGTTGGCTTATCGTGGTG and anti-sense primer: CTAACGAGGGGTCGATCTTGG. Real-time PCR was performed by using an iCycler iQ real-time PCR detection system (Bio-Rad, Hercules, CA). The mRNA levels were quantified as previously described [13]. Briefly, the cycle threshold (Ct) values of each gene of interest and of GAPDH were calculated for each sample and then the normalized value was derived by subtracting the Ct value of GAPDH from that of the gene of interest (∆Ct). Data are shown as mRNA fold change (2^-∆∆Ct^) relative to the mRNA level of the corresponding transcript in the control samples as indicated.

### Western blot

Cells were washed once with cold PBS, harvested by scraping, and lysed with RIPA Buffer containing a protease and phosphatase inhibitor cocktail (Thermo Scientific, Rockford, IL). Lysates (20 μg/lane) were loaded into each lane of an SDS–polyacrylamide gel; proteins were transferred onto a nitrocellulose membrane after size separation and analyzed by using specific antibodies. Anti-FKHR antibodies (H-128; sc-11350) and anti-GFP antibodies (sc-9996) were obtained from Santa Cruz Biotechnology (Santa Cruz, CA); anti-actin antibodies (A5441) were obtained from Sigma (St. Louis, MO); anti-CPT1A antibodies is a gift from Drs. Janos Kerner and Charles Hoppel [14].

### Soft agar colony formation assay

The soft agar colony formation assay was performed as previously described by us [12] and others [15], by using the CytoSelect 96-well Cell Transformation Kit (Cell Biolabs, San Diego, CA). Briefly, cells were incubated 8 days in a semisolid agar media before being solubilized, lysed, and detected by using the patented CyQuant GR Dye and an EnVision plate reader (PerkinElmer, Waltham, MA).

### Transient transfections of siRNAs and plasmids

A PAX3-FKHR–specific siRNA (PF siRNA) and control non-targeting siRNA (NT siRNA) were synthesized as described previously [11]. Pooled CPT1A siRNA (ON-TARGETplus SMARTpool L-009749-00-0005), individual CPT1A siRNA 1 (J-009749-07-0005), and individual CPT1A siRNA 2 (J-009749-09-0005) were obtained from Thermo Scientific (Thermo Scientific, Chicago, IL). Cells were plated in a 24-well plate 24 hours prior to transfection with siRNAs (10 nM) by using lipofectamine RNAiMAX reagent (Invitrogen), or plasmids by using FuGENE 6 (Invitrogen) according to manufacturer’s instructions. For cell growth, cells were monitored continuously after transfection. Wound healing assays were performed 24 hours after transfection. For evaluation of expression levels, cells were harvested 48 hour post-transfection.

### Scratch wound-healing and cell growth assays

The wound healing assay was performed to monitor and quantify cell motility. Briefly, cells were seeded in a 24-well plate (Essen ImageLock plate) at 1 × 10^5^ cells per well and allowed to reach confluence before the surface was uniformly scratched across the center of well by using a Essen wound maker provided by IncuCyte. The wells were then rinsed with fresh media to remove floating cells, and the wound healing process was monitored continuously by using the IncuCyte live-cell imaging system (Essen BioScience, Ann Arbor, Michigan). Images were obtained at each set time point and then analyzed to quantify wound healing by using the IncuCyte scratch wound assay software according to the manufacturers’ instructions. Data were expressed as % wound confluence as defined by the integrated metrics used to quantify the wound healing in the CellPlayer Cell Migration Assay of IncuCyte [16,17]. The CPT1A inhibitor Etomoxir was obtained from Sigma. Cell growth was measured also by using the IncuCyte system [18]. Briefly, cells were plated into 96-well or 24-well plate and allowed to grow for 24 hours with or without subsequent transfection, before acquiring phase-contrast images and an integrated confluence metric as a surrogate for cell number every 4 hours by IncuCyte.

### Luciferase reporter assay

Three tandem copies of each CPT1A putative PAX3-FKHR binding site, either wild-type (ie, PRO1, PRO2, PRO3, and PRO4) or with the corresponding PD site mutated (ie, PRO1m, PRO2m, PRO3m, and PRO4m), were placed upstream of a minimal thymidine kinase (tk) promoter [12] in the pGL4.20 reporter construct (Promega, Madison, WI). The resulting oligonucleotide sequences were as follows: PRO1, 5′-attacctgcctctctcgttctccttcattacctgcctctctcgttctccttcattacctgcctctctcgttctccttc-3′;PRO1m,5′-attacctgcctctctcatttaccttcattacctgcctctctcatttaccttcattacctgcctctctcatttaccttc-3′; PRO2, 5′-gttactcatcttcattacaactgttactcatcttcattacaactgttactcatcttcattacaac-3′; PRO2m, 5′-atttatcatcttcattacaactatttatcatcttcattacaactatttatcatcttcattacaac-3′; PRO3, 5′-gttctctgcttctttattaatagagttctctgcttctttattaatagagttctctgcttctttattaataga-3′; PRO3m,5′-atttactgcttctttattaatagaatttactgcttctttattaatagaatttactgcttctttattaataga-3′;PRO4,5′-attatgttcttgacgctggaagaattaattatgttcttgacgctggaagaattaattatgttcttgacgctggaagaatta-3′;PRO4m,5′-attatatttatgacgctggaagaattaattatatttatgacgctggaagaattaattatatttatgacgctggaagaatta-3′.

NIH3T3 cells were co-transfected with pcDNA3 or pcDNA3-PAX3-FKHR [11], one of the firefly luciferase reporters (pGL4.20-PRO) described above, and a constitutively-expressed Renilla luciferase reporter (PRL-TK, control for transfection efficiency) (Promega) by using FuGENE 6 according to the manufacturer’s instructions. Then, 24 hours after transfection, 10 000 cells were plated in each well of a 96-well culture plate and grown for an additional 24 hours before luciferase activity was measured by using the Dual-Glo Luciferase Assay System (Promega) according to the manufacturer’s instructions. The firefly luciferase activity was normalized to that of Renilla luciferase.

### Electrophoretic mobility shift assay (EMSA)

EMSA was performed by using either nuclear extract or *in vitro*–translated protein. To prepare nuclear extract, HEK293T cells were transfected with either GFP–PAX3-FKHR–expressing vector [12] or GFP control vector. Transfected cells were harvested 48 hours later, and nuclear extracts were prepared by using the NE-PER* Nuclear and Cytoplasmic Extraction Kit (Thermo Scientific). To prepare *in vitro*–translated protein, pcDNA3-PAX3-FKHR was used to synthesize PAX3-FKHR protein by using TNT-Coupled Wheat Germ Extract Systems (Promega). EMSA was performed by using the LightShift Chemiluminescent EMSA kit (Thermo Scientific) according to the manufacturer’s instructions. Briefly, 3 μL of nuclear extract or *in vitro*–translated protein was incubated at room temperature for 20 min in a 20-μL volume containing 2.5% glycerol, 5 mM MgCl_2_, 50 ng/μL Poly (dI.dC), 0.05% NP-40, and biotin end-labeled CPT1A PRO2 with or without unlabeled PRO2 or PRO2m as indicated. Complexes were resolved using electrophoresis through a 5% native polyacrylamide gel.

### Microarray

Untransfected parental Rh30 cells (WT), Rh30 control clone (CON) and Rh30 clone with PAX3-FKHR stably knocked down (KD1) were used to identify transcriptional targets of PAX3-FKHR in the microarray assays. Total RNAs were amplified and labeled by using an Agilent Quick Amp labeling kit (part number 5190–0444, Santa Clara, CA) and following the two color protocol (v.5.7). We used the Agilent 4 × 44 K whole human genome oligo microarray (G4112F, AMADID #014850) that contains 45,015 features representing about 41,000 unique probes. Microarrays were scanned by using an Agilent scanner (G2565CA) at 3 μm resolution, and data were extracted by Agilent Feature Extraction software (v. 10.5.1.1). Lowess normalization on background-subtracted signal intensity was performed to correct the intensity bias, and dye bias was further corrected by a pair of dye swap experiments for each comparison. A list of differentially expressed genes (see Additional file 2: Table S1) were selected according to the following criteria: 1) an average of at least 2-fold expression change was detected in 2 comparisons (KD1 vs. WT and KD1 vs. CON), each with 2 biological replicates including a pair of dye-swap experiments; 2) the observed signal intensity was greater than the value of the 99th percentile of those of the negative controls in at least one channel; and 3) the differential expression was consistent between the dye-swap pairs in all comparisons.

### Statistical analysis

Results are expressed as the mean ± SD of at least 3 independent experiments. The Student’s *t*-test was used to determine the statistical significance of the difference between the paired samples. Differences were considered significant if *p* < 0.05 (*), 0.01 (**) or 0.001 (***) and non-significant (ns) if *p* > 0.05. Where applicable, sample pairs were noted in brackets.

## Results

### PAX3-FKHR promotes cell motility in ARMS cells

**Figure 1:**
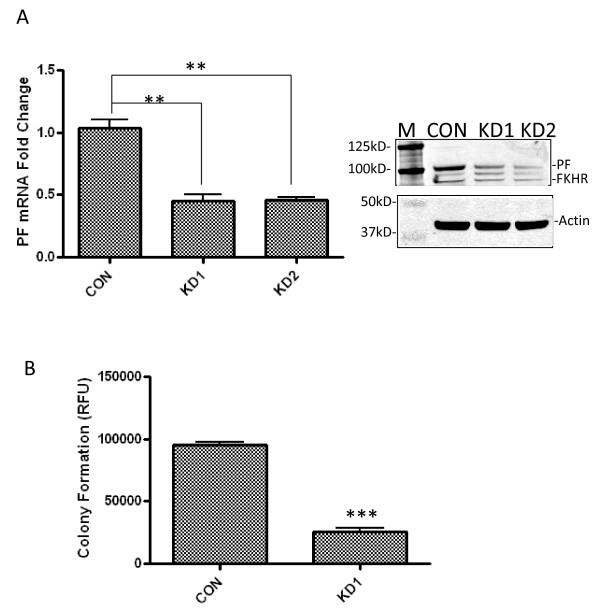
**Downregulation of PAX3-FKHR decreases anchorage-independent growth of Rh30 cells.** (**A**) shRNA against PAX3-FKHR downregulates the level of PAX3-FKHR. Rh30 cells stably transfected with either shRNA against PAX3-FKHR (KD1 and KD2, two different clones generated from the same shRNA) or a control non-targeting shRNA (CON) were used to analyze PAX3-FKHR mRNA or protein levels by using real-time RT-PCR (left panel) or Western blotting (right panel), respectively. GAPDH was used as an internal control for RT-PCR experiments, and PAX3-FKHR mRNA levels in KD1 and KD2 were normalized to CON (PF mRNA fold-change). Actin was the equal loading control for Western blot experiments. M, molecular weight marker; PF, PAX3-FKHR (~99kD); FKHR (~70kD) was also indicated. (**B**) Downregulation of PAX3-FKHR decreases anchorage-independent growth of Rh30. Raw fluorescent units (RFUs) were used to represent colony numbers of Rh30 control clone (CON) and KD1; and the average of triplicate measurements is shown.

To study the role of PAX3-FKHR in regulating ARMS cell growth and motility, PAX3-FKHR was stably knocked down in human ARMS cell line Rh30. As shown in Figure 1A, both mRNA (left panel) and protein levels (right panel) of PAX3-FKHR in the knockdown clones (KD1 and KD2, two stable clones generated from the same RNAi) were noticeably downregulated compared to a control clone (CON). In agreement with previously reported results [2], downregulation of PAX3-FKHR decreased the rate of cell growth ( Additional file 1: Figure S1; KD1 and KD2 have the same growth rate); the transformation potential of these cells was decreased by 73%, as observed in a soft agar colony formation assay (Figure 1B).

**Figure 2:**
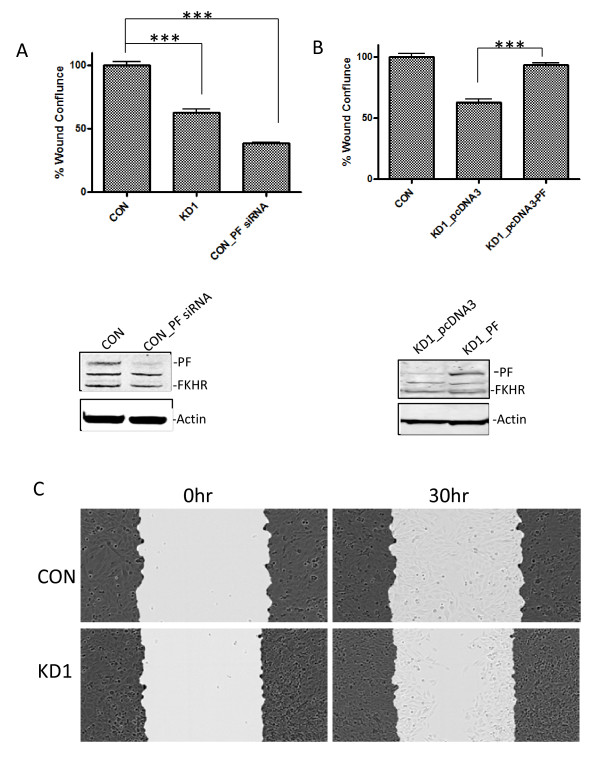
**Downregulation of PAX3-FKHR decreases the motility of Rh30 cells.** (**A**) Motility of Rh30 cells control clone (CON) decreases when PAX3-FKHR is either stably knocked down (KD1) or transiently knocked down (CON_PF siRNA). The protein levels of PF were shown below the bar graph. The wound confluence of CON was arbitrarily set as 100%. **(B)** The decreased cell motility of KD1 can be rescued by transiently expressing pcDNA3-PAX3-FKHR (KD1_pcDNA3-PF) but not by expressing an empty vector (KD1_pcDNA3). The protein levels of PF in KD1 transfected with pcDNA3 or pcDNA3-PF were shown below the bar graph. Rh30 control clone (CON) was shown as a reference. PAX3-FKHR siRNA (PF siRNA) or plasmids (either pcDNA3 or pcDNA3-PF) were transfected into CON (CON_PF siRNA) in (A) or KD1 (pcDNA3 or pcDNA3-PF) in (B) 24 hours before real-time monitoring of wound healing. The data, expressed as % Wound Confluence, represent the quantitation of wound healing 30 hours after the scratch was created. (**C**) Representative microscopic images taken 30 hours after the scratch was created.

To evaluate the effect of downregulating PAX3-FKHR on cell motility, we used a wound-healing assay. As shown in Figure 2A, downregulating PAX3-FKHR, either stably (KD1), or transiently (CON_PF_siRNA) by using a specific siRNA against PAX3-FKHR (PF siRNA), decreased cell motility compared to that of the Rh30 control clone (CON) by 37% and 61%, respectively. The decreased cell motility in the PAX3-FKHR knocked-down clone (KD1) can be rescued by transiently introducing PAX3-FKHR ectopically back into the Rh30 KD clone (Figure 2B). Figure 2C shows representative microscopic images of newly created wounds in Rh30 control clone and KD1 clone cells and the repaired wounds 30 hours later. Similar observations were made when KD2 was used (data not shown). Our observations are consistent with previous studies using either Rh4 [19] or Rh18 ARMS cells [20], confirming that PAX3-FKHR regulates ARMS cell proliferation, transformation potential and motility. Importantly, our data indicate that Rh30 and its PAX3-FKHR knocked-down clone (KD1) is an appropriate pair of cell models for the studies of PAX3-FKHR function, including transcriptional regulation of its target genes.

### PAX3-FKHR regulates the expression of CPT1A in ARMS cells

We hypothesized that the defective cell motility in KD1 was caused by the decreased expression of one or more of PAX3-FKHR’s target genes. To identify the target genes of PAX3-FKHR, we compared the mRNA profiles in KD1 with those in either Rh30 or CON by using microarray analysis (Geo no. GSE35862; also see Methods section for a detailed description of selection criteria). Among the 126 genes with at least 2-fold expression change, 96 were downregulated in KD1 clone (see Additional file 2: Table S1), including cannabinoid receptor 1 (*CNR1*), which has been previously reported as a PAX3-FKHR target gene in multiple studies [1], and *ALDH1A3, EYA2, MCTP2, MXRA5, NTF3, OLIG1, SEPP1, SULF2,* and *TOX3*, which were recently identified as PAX3-FKHR target genes in ARMS cell line Rh4 [21].

**Figure 3:**
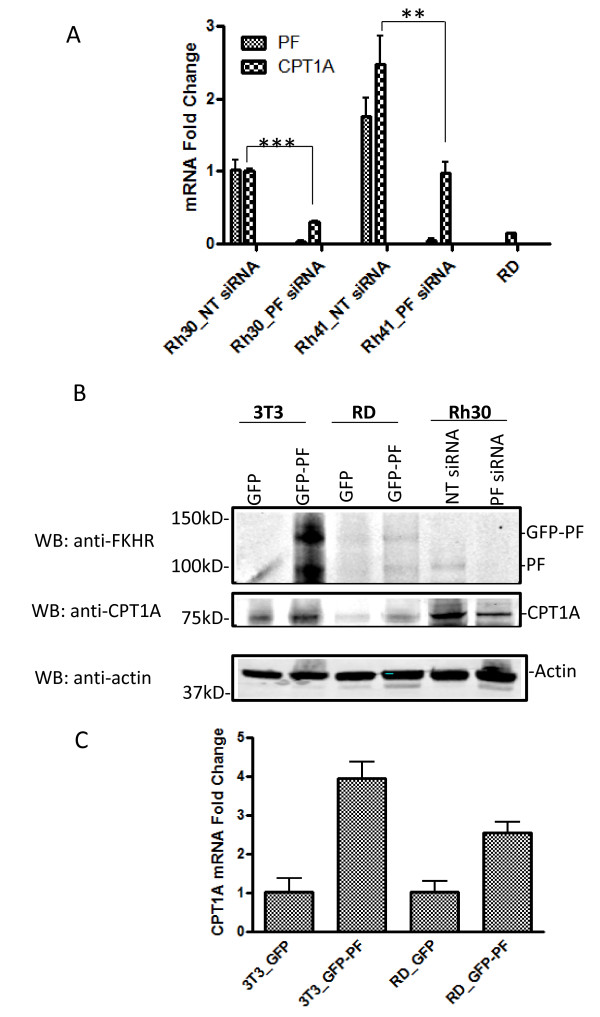
**PAX3-FKHR regulates the expression of CPT1A.** Levels of CPT1A and PAX3-FKHR mRNA were determined by using quantitative real time RT-PCR or Western blot. (**A**) The mRNA levels of PAX3-FKHR or CPT1A in control Rh30 cells was arbitrarily set as 1, and the mRNA levels of PAX3-FKHR or CPT1A measured under other experimental conditions were expressed as “mRNA fold change” by normalizing them to the corresponding mRNA level in Rh30 cells. Rh30_PF siRNA, Rh30 cells transiently transfected with PF siRNA; Rh41_PF siRNA, Rh41 transiently transfected with PF siRNA. Rh30_NT siRNA, Rh41_NT siRNA, and RD represents Rh30, Rh41, and RD cells transiently transfected with NT siRNA, respectively. (**B**) Protein levels of PAX3-FKHR (GFP-PF and PF) and CPT1A in cells (NIH3T3 or RD) transiently transfected with either GFP or GFP-PF, or Rh30 transiently transfected with either NT siRNA or PF siRNA. Actin was an equal loading control. (**C**) RD or NIH3T3 cells were transiently transfected with either GFP empty vector or GFP-PAX3-FKHR (GFP-PF). The mRNA levels of CPT1A in 3T3/GFP or RD/GFP was arbitrarily set as 1.

CPT1A was identified as a gene whose expression in KD1 is significantly downregulated (an average of 8-fold reduction) compared to that in wild-type Rh30 cells or Rh30 control clone cells. To confirm that the reduced CPT1A expression was correlated to the decreased PAX3-FKHR expression, Rh30 cells were transiently transfected with PF siRNA; the downregulation of PAX3-FKHR (97% reduction at mRNA level) led to a downregulation of CPT1A mRNA (70% reduction) (Figure 3A) and protein levels (Figure 3B). Similar results were observed in another ARMS cell line, Rh41 (Figure 3A). Additionally, both the mRNA (Figure 3A) and protein levels (Figure 3B) of CPT1A are very low in RD, a PAX3-FKHR–negative human ERMS cell line. When PAX3-FKHR is transfected into either RD or NIH3T3 cells, CPT1A levels are moderately induced (Figure 3B and 3C). These results suggested that CPT1A might be a transcriptional target of PAX3-FKHR.

### Induction of CPT1A transcription by PAX3-FKHR involves a paired-box domain (PD) binding site-dependent transactivation

**Figure 4:**
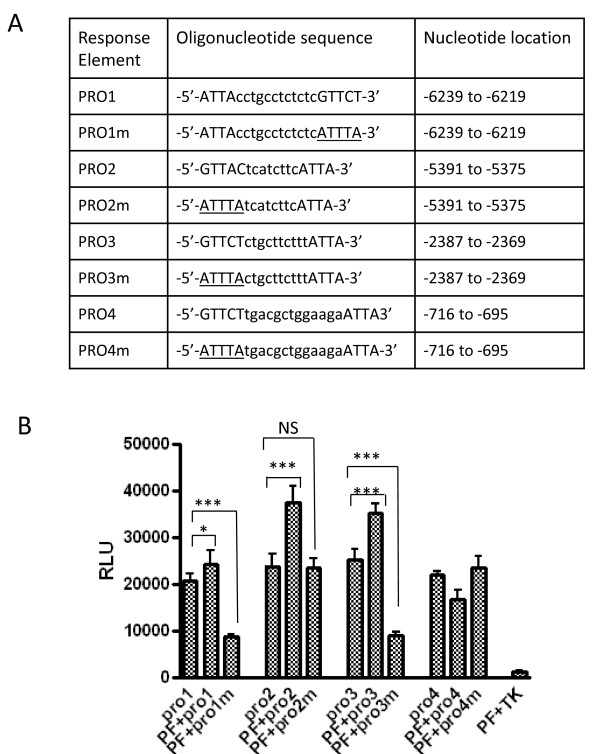
**Functional analysis of the putative PAX3-FKHR response elements upstream of the*CPT1A*transcription start site (TSS).** (**A**) The putative binding sites (ie, PRO1, PRO2, PRO3 and PRO4) containing a composite PD (GTTC/AT/C) and HD (ATTA) recognition site and the corresponding mutants with the PD site mutated (ie, PRO1m, PRO2m, PRO3m and PRO4m). Nucleotide locations are relative to the TSS. (**B**) Transcriptional activity of putative PAX3-FKHR binding sites. NIH3T3 cells were transfected as indicated 24 hours prior to luciferase assays. TK is the reporter without any PRO sequence and was used as a negative control. Data were expressed as relative luciferase units (RLUs) by normalizing firefly luciferase activity to Renilla luciferase activity. PF, PAX3-FKHR.

To investigate whether PAX3-FKHR controls *CPT1A* transcription by binding to *CPT1A*’s promoter, we analyzed the DNA sequence 5′ to the *CPT1A* transcription start site and identified 3 putative PAX3-FKHR binding sites that matched the consensus composite sequence: a paired-box domain (PD) binding site (GTTC/AT/C) followed by a linker sequence and a homeodomain (HD) binding site (ATTA) (Figure 4A). For comparison, we also included PRO1, a sequence that contains an HD binding site followed by a PD binding site in our analysis. To investigate the contribution of these putative PAX3-FKHR binding sites to the transactivation of *CPT1A*, we generated 4 luciferase reporter constructs: PRO1-tk-luc, PRO2-tk-luc, PRO3-tk-luc and PRO4-tk-luc (Figure 4A and B). In NIH3T3 cells, a PAX3-FKHR–negative cell line commonly used to evaluate the function of PAX3-FKHR [22], ectopic expression of PAX3-FKHR substantially transactivated PRO2-tk-luc (57% increase) and PRO3-tk-luc (42% increase), only slightly transactivated PRO1-tk-luc (17% increase), and failed to transactivate PRO4-tk-luc, suggesting that PRO2, PRO3, and possibly PRO1 might contain the binding element for PAX3-FKHR. To confirm this, we mutated the putative PD-binding site in each reporter construct to generate PRO1m-tk-luc, PRO2m-tk-luc, PRO3m-tk-luc, and PRO4m-tk-luc (Figure 4A) and then tested the responses of each to PAX3-FKHR expression. As shown in Figure 4B, mutating the PD-binding site in PRO2 abolished its transactivation by PAX3-FKHR. Mutating the PD-binding site in PRO1 and PRO3 not only abolished the reporters’ transactivation by PAX3-FKHR but also decreased their basal activities (65% decrease for PRO1 and 69% decrease for PRO3), possibly because the basal activation of PRO1 and PRO3 is primarily mediated by mechanism independent of PAX3-FKHR. The requirement of an intact PD binding site for PAX3-FKHR–mediated transactivation is consistent with previous reports [5,21,23]. Our data suggest that the promoter region of *CPT1A* contains the binding sites for PAX3-FKHR, which are required for PAX3-FKHR-mediated transactivation.

### PAX3-FKHR participates in regulating CPT1A transcription

**Figure 5:**
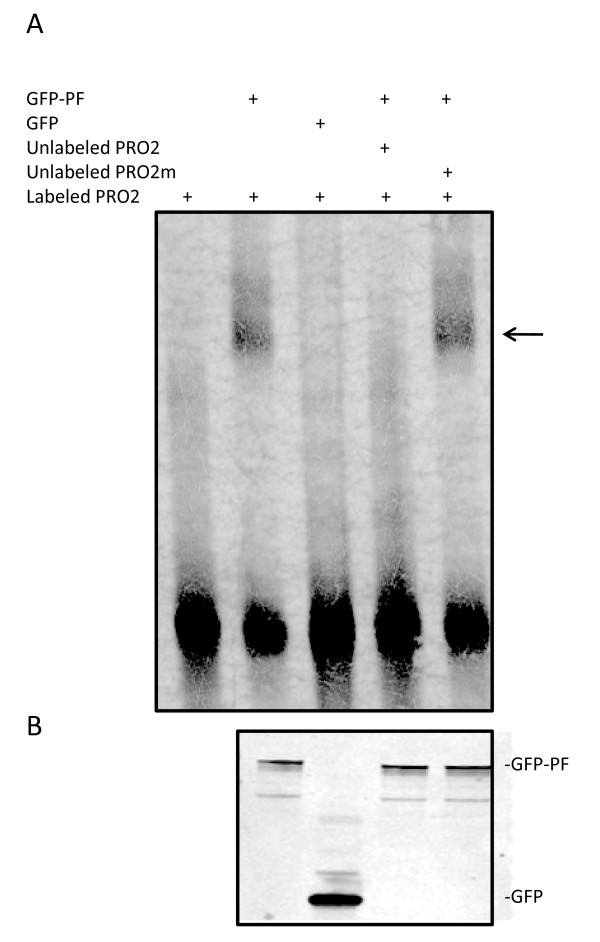
**PAX3-FKHR binds to the*CPT1A*promoter.** (**A**) EMSA analysis of GFP–PAX3-FKHR binding to CPT1A putative binding site 2 (PRO2). All lanes contain labeled PRO2 in addition to other components, as indicated. 100-fold excess unlabeled wild-type (PRO2) or PD-binding site mutant (PRO2m) was used to compete with the labeled PRO2 DNA probe for GFP-PAX3-FKHR binding. GFP-PF, nuclear extract prepared from HEK293T cells transfected with GFP-PAX3-FKHR; GFP, nuclear extract prepared from HEK293T cells transfected with GFP vector only. Arrow indicates the PAX-FKHR-DNA complexes. (**B)** The amount of GFP-PF and GFP in the nuclear extracts used for the EMSA, revealed by using an anti-GFP antibody in a Western blot analysis.

We next investigated whether PAX3-FKHR interacts with the promoter region of *CPT1A* by performing an *in vitro* electrophoretic mobility shift assay (EMSA). As shown in Figure 5A, biotin-labeled wild-type PRO2 probe (lane 1) was shifted by the addition of nuclear extract from HEK293T cells transfected with GFP-PAX3-FKHR (lane 2), but not by nuclear extract from HEK293T cells transfected with a control vector (GFP only) (lane 3), indicating the formation of a specific complex between GFP–PAX3-FKHR and the biotin-labeled wild-type PRO2. The specific DNA-protein complex was disrupted by the inclusion of a wild-type un-labeled PRO2 (lane 4), but not by a mutated un-labeled PRO2m (lane 5). The amount of GFP–PAX3-FKHR and GFP used in the EMSA assay is shown in Figure 5B. Similar results were obtained when *in vitro*-translated PAX3-FKHR was used ( Additional file 3: Figure S2). These results indicate that PAX3-FKHR binds to the promoter region of *CPT1A* in a PD binding site–dependent manner, further confirming that *CPT1A* is a transcriptional target of PAX3-FKHR.

### CPT1A promotes cell motility in ARMS cells

**Figure 6:**
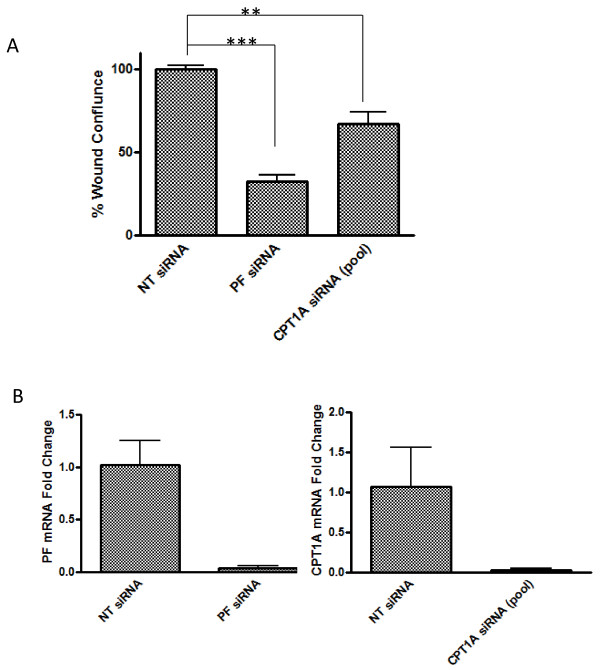
**Downregulation of CPT1A decreases cell motility in Rh30 cells.** (**A**) NT siRNA, PAX3-FKHR siRNA, or CPT1A siRNA (ON-TARGETplus SMARTpool) was transfected into Rh30 cells, and wound healing assays were performed as described in Figure 2. (**B**) The knockdown efficiency was confirmed by using real-time RT-PCR.

**Figure 7:**
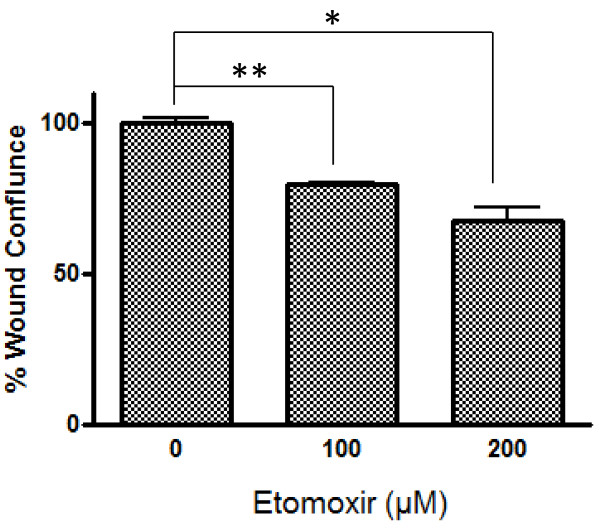
**Inhibition of CPT1A decreases the motility of Rh30 cells.** Wound healing assay was performed as described in Figure 2, with or without Etomoxir (100 or 200 μM).

To determine whether PAX3-FKHR’s regulation of motility in ARMS cells is mediated by CPT1A, we first measured the effect of downregulating *CPT1A* on Rh30 cell motility in the wound-healing assay. *CPT1A* siRNA reduced Rh30 cell motility by 33% (Figure 6A), whereas PF siRNA reduced it by 67%. *CPT1A* siRNA has less effect on wound-healing than PF siRNA, possibly because other transcription targets of PAX3-FKHR also play roles in regulating cell motility. The high efficiency of siRNA-mediated CPT1A and PAX3-FKHR downregulation is shown in Figure 6B. The effect of *CPT1A* siRNA on cell motility was confirmed by using two different individual siRNAs, *CPT1A* siRNA 1 and *CPT1A* siRNA 2 ( Additional file 4: Figure S3A and S3B). Whereas *CPT1A* siRNA significantly reduced Rh30 cell motility, its effect on the cell growth of Rh30 is marginal ( Additional file 4: Figure S3C). We further confirmed the effect of downregulating *CPT1A* on cell motility by using Etomoxir, a pharmacologic inhibitor of CPT1 [24]. In published studies, etomoxir were used at 100 – 200 μM range in cell-based assays [24]. We tested a range of etomoxir concentrations between 10 and 200 μM. Etomoxir decreased the motility of Rh30 cells in a dose-dependent manner (Figure 7 and Additional file 5: Figure S4A). The effect of etomoxir on cell motility was significant only at higher concentration ( Additional file 5: Figure S4A), possibly because of the high level of CPT1A present in Rh30 cells (Figure 3B). However, the effect of etomoxir is specific to Rh30 cells – at all concentrations tested, etomoxir failed to reduce the cell motility of RD ( Additional file 5: Figure S4B), a PAX3-FKHR-negative ERMS cell line with very low level of CPT1A (Figure 3A and 3B). These results indicate that downregulation of CPT1A, either by gene knockdown, or by pharmacologic intervention, leads to decreased cell motility in Rh30 cells. Taken together, these data reveal a novel function of CPT1A in promoting cell motility in ARMS.

## Discussion

Intensive efforts have recently been made to identify the signaling pathways involved in PAX3-FKHR–mediated ARMS tumorigenesis, including efforts to identify the transcription targets of PAX3-FKHR [1]. The rationale of our study is that if the transcription targets of PAX3-FKHR also mediate the function of PAX3-FKHR in regulating ARMS tumorigenesis, then these PAX3-FKHR transcription targets would be novel therapeutic targets for ARMS. However, few genes have been commonly identified by previous studies using different approaches [25]. Some studies focused on induction of gene expression by overexpressing PAX3-FKHR in a non-ARMS background, however, ectopic expression of PAX3-FKHR might not lead to induction of endogenous PAX3-FKHR target genes in a non-ARMS environment. Other studies focused on genes whose expression is present in PAX3-FKHR-positive tumor samples but is absent in PAX3-FKHR–negative tumor samples, however, PAX3-FKHR is not the only difference between these tumor samples [1,25]. In order to identify transcriptional targets of PAX3-FKHR in an ARMS cellular context, we used PAX3-FKHR-specific shRNA to stably knockdown PAX3-FKHR in Rh30 cells. In the PAX3-FKHR stable knockdown clones we used, only 50% of PAX3-FKHR was knocked down, likely because PAX3-FKHR positively regulates the cell growth of Rh30 cells; therefore, complete knockdown of PAX3-FKHR would significantly and negatively affect the growth of Rh30 cells. Indeed, even with only 50% stable knockdown of PAX3-FKHR in Rh30 cells, there is already a drastic defect in anchorage-independent growth. Similarly, the cell motility, as revealed by the wound healing assay, decreases significantly in the stable knockdown clones. The observations that PAX3-FKHR regulates ARMS cell growth and cell motility were in agreement with those made in previous studies of other ARMS cell lines [2,19,20]. Because our knockdown clones had significant defects in the function of PAX3-FKHR, we used them for gene-profiling analysis. Among the genes whose expression was downregulated in the PAX3-FKHR knockdown clone were *CNR1, ALDH1A3, EYA2, MCTP2, MXRA5, NTF3, OLIG1, SEPP1, SULF2,* and *TOX3* ( Additional file 2: Table S1), genes previously shown to be target genes of PAX3-FKHR in several independent studies [1,21], and *CPT1A*, a novel transcriptional target gene of PAX3-FKHR. *CPT1A* was not identified as a target gene of PAX3-FKHR in other studies, most likely because of the different cellular model systems used. Interestingly and significantly, downregulation of CPT1A causes defects in cell motility, at least partially recapitulating the defects caused by downregulation of PAX3-FKHR. In addition, overexpression of PAX3-FKHR in either RD or NIH3T3 cells leads to moderate upregulation of CPT1A. Furthermore, PAX3-FKHR binds to the promoter region of *CPT1A* in a PD binding site–dependent manner. Therefore, our study identified *CPT1A* as a novel transcriptional target of PAX3-FKHR that also carries out the function of PAX3-FKHR in regulating cell motility.

CPT1 is a key regulatory enzyme regulating fatty-acid oxidation and has been heavily studied as a therapeutic target for the treatment of the metabolic syndrome [26]. A few recent reports also implicate the involvement of CPT1 in regulating apoptosis and in cancer development [27,28]. Despite decreased expression in the cytoplasm of several tumor tissues, CPT1A expression was found to localize in the nuclei, where it interacts with histone deacetylase protein complexes in neoplastic cells [29]. A recent study showed that CPT1C is frequently upregulated in human lung tumor and that CPT1C depletion via siRNA suppresses xenograft tumor growth *in vivo*, suggesting that CPT1C may be a new therapeutic target for the treatment of hypoxic tumors [10]. Another report suggests that in prostate cancer, androgen upregulates the mRNA level of CPT1, leading to increased mitochondrial oxidation of fatty acids and increased production of reactive oxygen species known to be associated with prostate cancer cell proliferation [30]. The role of CPT1 in regulating cancer cell proliferation appears to be emerging. Our study identifies *CPT1A* as a transcriptional target of PAX3-FKHR. Furthermore, downregulation of CPT1A at least partially mimics the effect of downregulating PAX3-FKHR, providing a functional link between PAX3-FKHR and CPT1A in regulating cell motility in ARMS. This is the first evidence that CPT1A positively contributes to the regulation of cancer cell motility.

## Conclusions

In conclusion, our goal was to identify druggable transcription targets of PAX3-FKHR that are also downstream effectors of PAX3-FKHR–mediated cell motility. We provide evidences that CPT1A is such a transcriptional target of PAX3-FKHR that also regulates cell motility. Pharmacologic modulator of CPT1 is available, and CPT1 has been studied as a therapeutic target for the treatment of the metabolic syndrome [26]. Our study revealed the novel function of CPT1A in promoting cell motility, and suggests that CPT1A [4] could be a novel therapeutic target for ARMS. However, how CPT1A regulates cell motility is still unclear. Future efforts should be directed to better understand the molecular mechanism responsible for CPT1A-mediated cell motility, in order to facilitate the full validation of CPT1A as a novel therapeutic target for the treatment of ARMS.

## Competing interests

The authors declare that they have no competing interests.

## Authors’ contributions

LL carried out all experiments, performed data analysis, and drafted the manuscript. YW provided assistance in microarray study design and data analysis. JW provided assistance in EMSA. JC provided assistance in cell growth and wound healing analysis by using the IncuCyte system. TC conceived of the study, and participated in its design and coordination, revised and finalized the manuscript. All authors read and approved the final manuscript.

## Pre-publication history

The pre-publication history for this paper can be accessed here:

http://www.biomedcentral.com/1471-2407/12/154/prepub

## Supplementary Material

Additional file 1 **Figure S1. Downregulation of PAX3-FKHR slightly decreases the growth rate of Rh30 cells.** Cell growth of Rh30 control clone (CON) and PAX3-FKHR knockdown clones (KD1 and KD2) was monitored by using the IncuCyte live-cell imaging system, and was expressed as % of cell confluence as defined by the IncuCyte software. Click here for file

Additional file 2 Table S1. Genes downregulated when PAX3-FKHR is downregulated. Click here for file

Additional file 3 **Figure S2. *In vitro*EMSA analysis of PAX3-FKHR binding to CPT1A putative binding site 2 (PRO2).** All lanes contain labeled PRO2 in addition to other components, as indicated. 100-fold excess unlabeled wild-type (PRO2) or PD-binding site mutant (PRO2m) was used to compete with the labeled PRO2 DNA probe for PAX3-FKHR binding. PF*, in vitro translated* PAX3-FKHR. Arrow indicates the PAX3-FKHR-PRO2 complexes. The amount of PAX3-FKHR used for the EMSA was shown in the lower panel in a Western blotting analysis using anti-FKHR antibodies. Click here for file

Additional file 4 **Figure S3. Downregulation of CPT1A decreases cell motility in Rh30 cells.** (A) Individual CPT1A siRNA (1 or 2) was transiently transfected into Rh30 cells. Wound healing assays were performed as described in Figure 2. (B) The knockdown efficiency was revealed by using real-time RT-PCR. (C) CPT1A siRNA does not significantly affect cell growth. Click here for file

Additional file 5 **Figure S4. Etomoxir decreases the motility of Rh30, but not RD cells.** Rh30 (A) or RD (B) cells were treated with different concentration of etomoxir and cell motility was monitored at different time points as indicated. Wound healing assays were performed as described in Figure 2. Click here for file
