# Supplementary figures and images for: Carnitine palmitoyltransferase 1A (CPT1A): a transcriptional target of PAX3-FKHR and mediates PAX3-FKHR–dependent motility in alveolar rhabdomyosarcoma cells
Source: BMC Cancer. 2012 Apr 25;12:154. doi: 10.1186/1471-2407-12-154 (PMC3453510; doi:10.1186/1471-2407-12-154)

## Slide 1
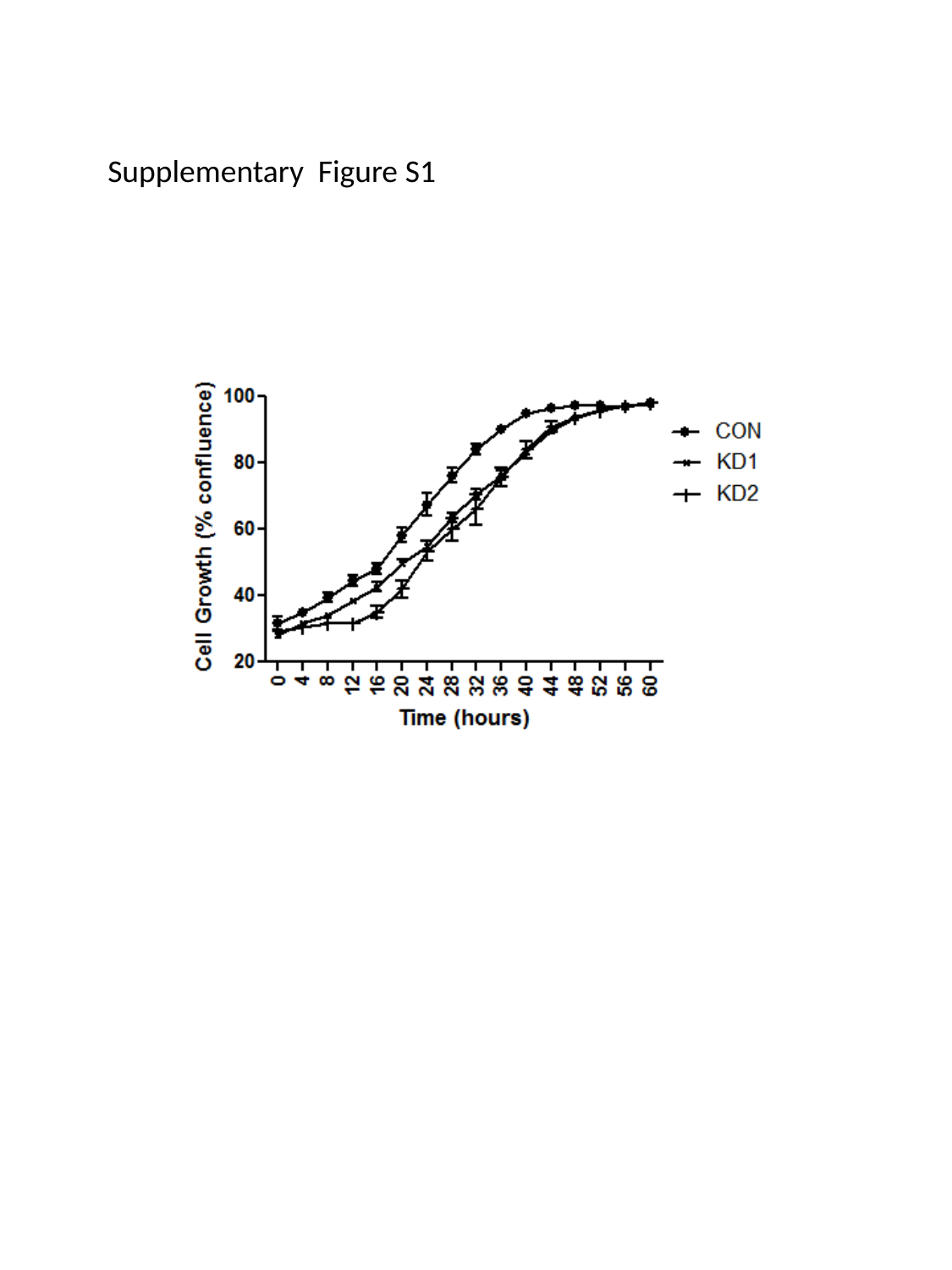

Supplementary Figure S1

Supplement: Additional file 1 — Figure S1. Downregulation of PAX3-FKHR slightly decreases the growth rate of Rh30 cells. Cell growth of Rh30 control clone (CON) and PAX3-FKHR knockdown clones (KD1 and KD2) was monitored by using the IncuCyte live-cell imaging system, and was expressed as % of cell confluence as defined by the IncuCyte software. [file 1471-2407-12-154-S1.ppt]

## Slide 1
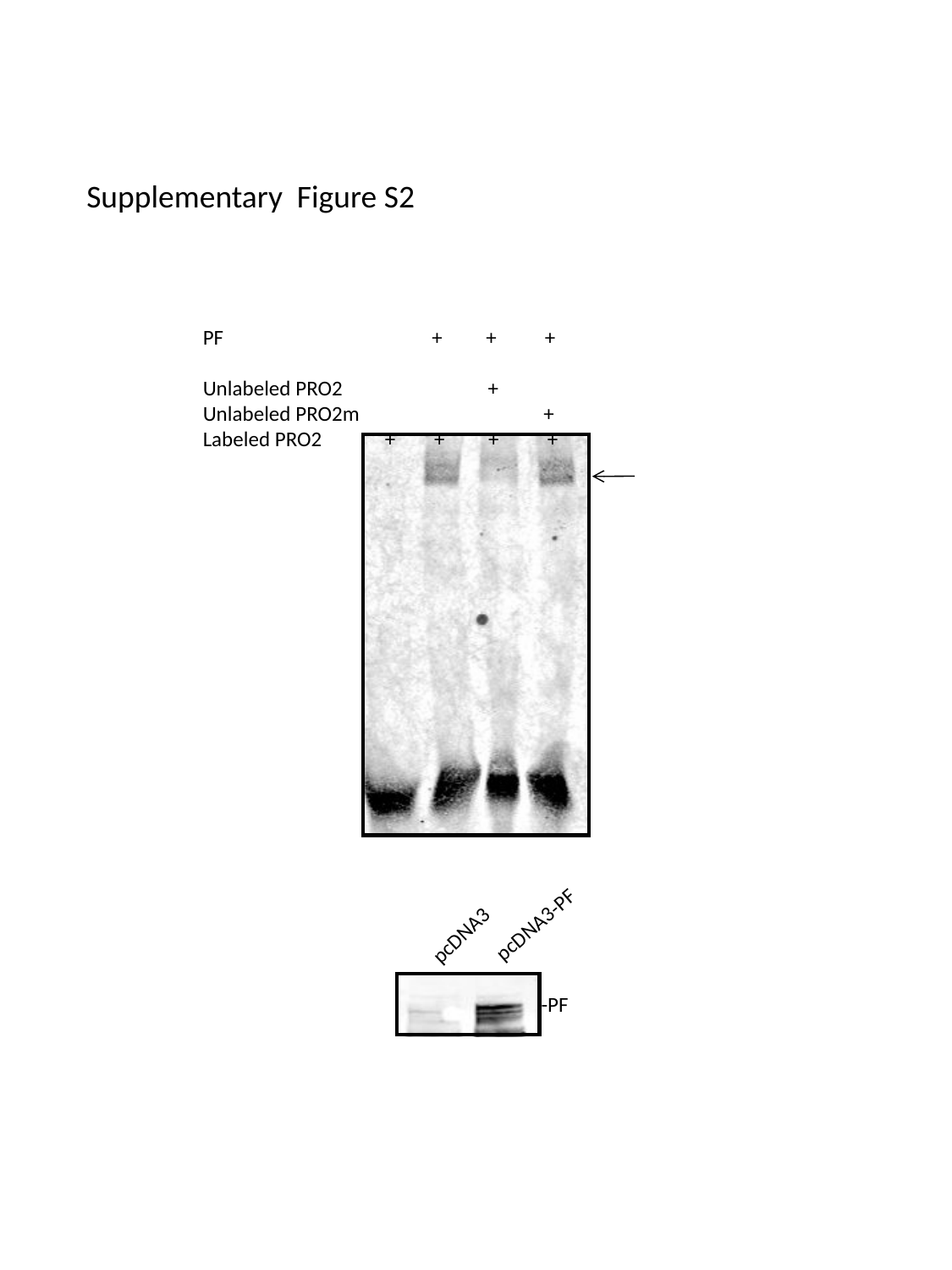

Supplementary Figure S2
PF + + +
Unlabeled PRO2 +
Unlabeled PRO2m +
Labeled PRO2 + + + +
pcDNA3
pcDNA3-PF
-PF

Supplement: Additional file 3 — Figure S2. In vitroEMSA analysis of PAX3-FKHR binding to CPT1A putative binding site 2 (PRO2). All lanes contain labeled PRO2 in addition to other components, as indicated. 100-fold excess unlabeled wild-type (PRO2) or PD-binding site mutant (PRO2m) was used to compete with the labeled PRO2 DNA probe for PAX3-FKHR binding. PF, in vitro translated PAX3-FKHR. Arrow indicates the PAX3-FKHR-PRO2 complexes. The amount of PAX3-FKHR used for the EMSA was shown in the lower panel in a Western blotting analysis using anti-FKHR antibodies. [file 1471-2407-12-154-S3.ppt]

## Slide 1
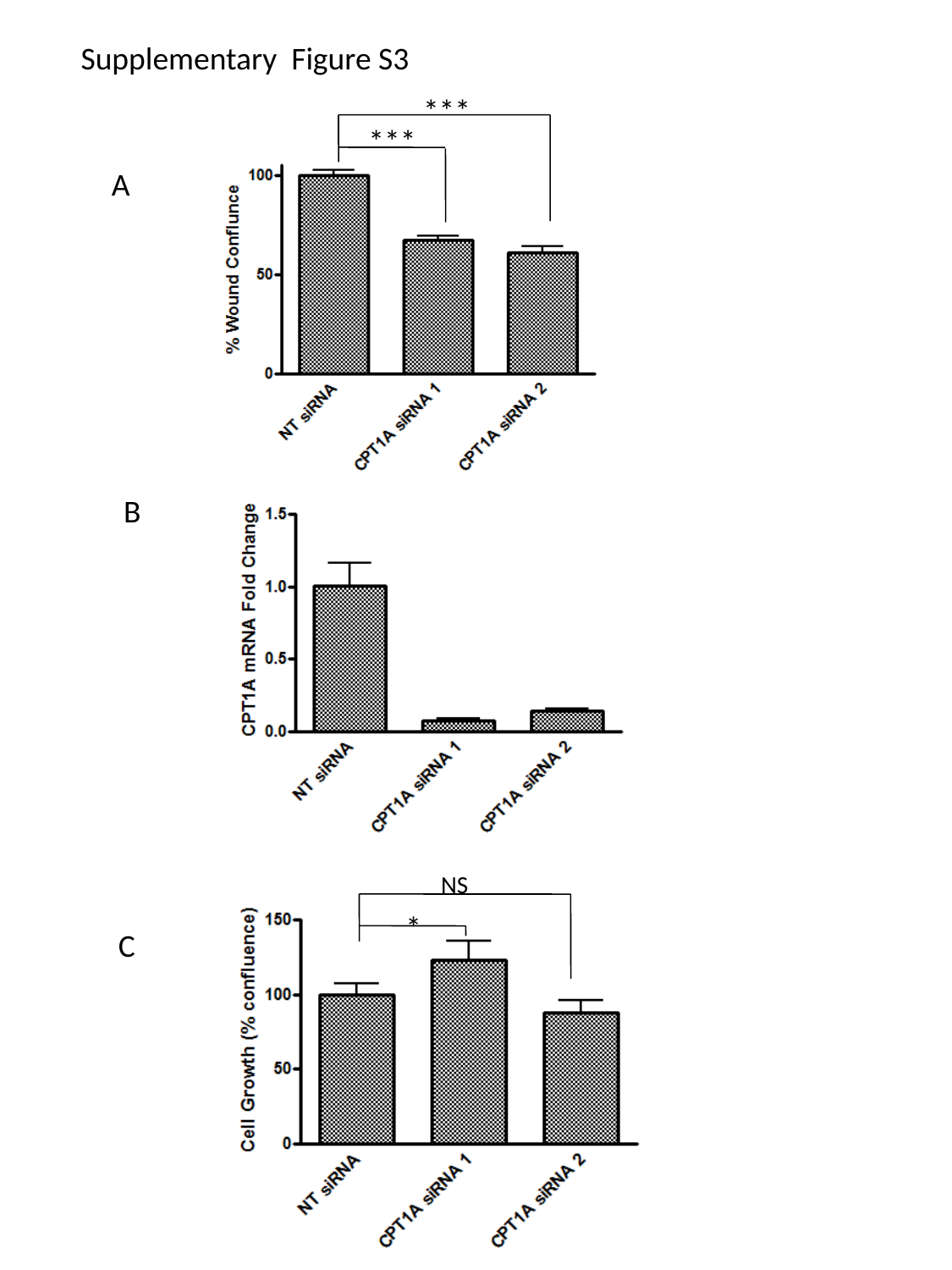

Supplementary Figure S3
***
***
A
B
NS
*
C

Supplement: Additional file 4 — Figure S3. Downregulation of CPT1A decreases cell motility in Rh30 cells. (A) Individual CPT1A siRNA (1 or 2) was transiently transfected into Rh30 cells. Wound healing assays were performed as described in Figure 2. (B) The knockdown efficiency was revealed by using real-time RT-PCR. (C) CPT1A siRNA does not significantly affect cell growth. [file 1471-2407-12-154-S4.ppt]

## Slide 1
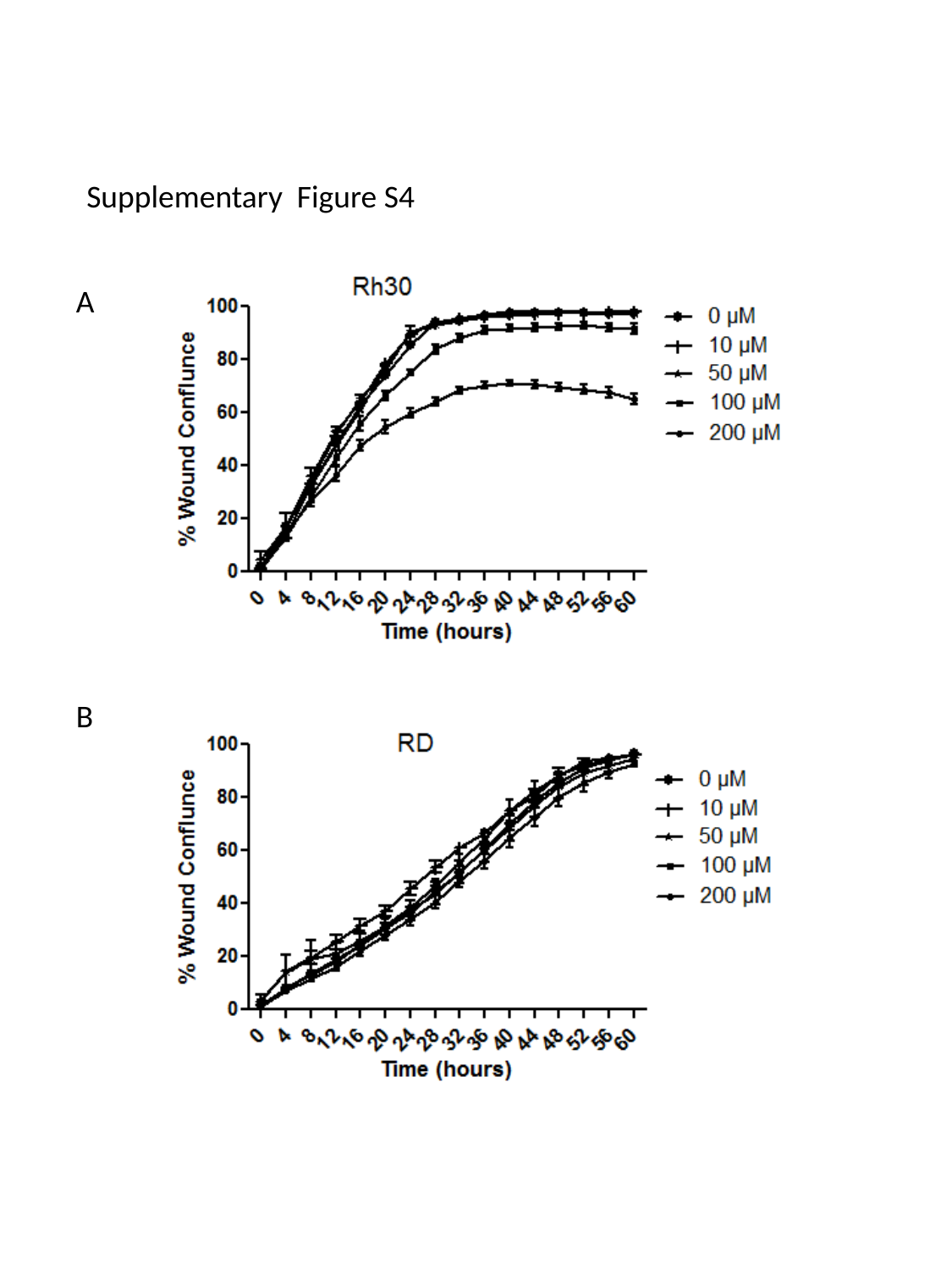

Supplementary Figure S4
A
B

Supplement: Additional file 5 — Figure S4. Etomoxir decreases the motility of Rh30, but not RD cells. Rh30 (A) or RD (B) cells were treated with different concentration of etomoxir and cell motility was monitored at different time points as indicated. Wound healing assays were performed as described in Figure 2. [file 1471-2407-12-154-S5.ppt]
