# Supplementary material for: Carnitine palmitoyltransferase 1A (CPT1A): a transcriptional target of PAX3-FKHR and mediates PAX3-FKHR–dependent motility in alveolar rhabdomyosarcoma cells
Source: BMC Cancer. 2012 Apr 25;12:154. doi: 10.1186/1471-2407-12-154 (PMC3453510; doi:10.1186/1471-2407-12-154)
Supplement: Additional file 2 — Table S1. Genes downregulated when PAX3-FKHR is downregulated. [file 1471-2407-12-154-S2.doc]

**Supplementary Table S 1. Genes downregulated when PAX3-FKHR is downregulated**

| **Symbol** | **Accession Number** | **Full Gene Name** |
| --- | --- | --- |
| C9orf125 | NM_032342 | chromosome 9 open reading frame 125 |
| TUSC1 | NM_001004125 | tumor suppressor candidate 1 |
| C5orf62 | NM_032947 | chromosome 5 open reading frame 62 |
| LAYN | NM_178834 | layilin |
| NETO2 | NM_018092 | neuropilin (NRP) and tolloid (TLL)-like 2 |
| SUMO3 | NM_006936 | SMT3 suppressor of mif two 3 homolog 3 |
| ITPRIPL2 | NM_001034841 | inositol 1,4,5-triphosphate receptor interacting protein-like 2 |
| SPESP1 | NM_145658 | sperm equatorial segment protein 1 |
| BMPR1B | NM_001203 | bone morphogenetic protein receptor, type IB |
| BC034319 | BC034319 | cDNA clone IMAGE:4837650 |
| HEPH | NM_014799 | hephaestin, transcript variant 2 |
| MAGEH1 | NM_014061 | melanoma antigen family H |
| FLI1 | NM_002017 | Friend leukemia virus integration 1 |
| GPM6B | NM_001001996 | glycoprotein M6B, transcript variant 2 |
| OLIG1 | NM_138983 | oligodendrocyte transcription factor 1 |
| FBXO4 | NM_012176 | F-box protein 4 , transcript variant 1 |
| FBN2 | NM_001999 | fibrillin 2 |
| SIRPA | NM_001040022 | signal-regulatory protein alpha, transcript variant 1 |
| NTF3 | NM_002527 | neurotrophin 3, transcript variant 2 |
| NLRX1 | NM_170722 | NLR family member X1 |
| SEPP1 | NM_005410 | selenoprotein P, plasma, 1 (SEPP1), transcript variant 1 |
| C7orf46 | NM_001127364 | chromosome 7 open reading frame 46, transcript variant 2 |
| LOC401022 | BC030713 | hypothetical LOC401022, mRNA (cDNA clone IMAGE:4827714) |
| SULF2 | NM_018837 | sulfatase 2, transcript variant 1 |
| TBC1D8B | NM_017752 | TBC1 domain family, member 8B (with GRAM domain), transcript variant 1 |
| SFRS13B | NM_080743 | splicing factor, arginine/serine-rich 13B |
| ZNF512B | NM_020713 | zinc finger protein 512B |
| LGI2 | NM_018176 | leucine-rich repeat LGI family, member 2 |
| ARHGAP25 | NM_001007231 | Rho GTPase activating protein 25, transcript variant 1 |
| USP43 | NM_153210 | ubiquitin specific peptidase 43 |
| DLL3 | NM_203486 | delta-like 3 transcript variant 2 |
| PTPRE | NM_006504 | protein tyrosine phosphatase, receptor type, E (PTPRE), transcript variant 1 |
| RNASEL | NM_021133 | ribonuclease L (2',5'-oligoisoadenylate synthetase-dependent) |
| EYA2 | NM_005244 | eyes absent homolog 2 , transcript variant 1 |
| COL2A1 | NM_001844 | collagen, type II, alpha 1, transcript variant 1 |
| ATP2A3 | NM_174953 | ATPase, Ca++ transporting, ubiquitous, transcript variant 5 |
| CNR1 | NM_033181 | cannabinoid receptor 1, transcript variant 2 |
| CPT1A | NM_001031847 | carnitine palmitoyltransferase 1A |

| BC043547 | BC043547 | clone IMAGE:5171873 |
| --- | --- | --- |
| SYTL4 | NM_080737 | synaptotagmin-like 4, transcript variant 1 |
| ARRDC2 | NM_001025604 | arrestin domain containing 2, transcript variant 2 |
| ENST00000368503 | ENST00000368503 | Discoidin, CUB and LCCL domain-containing protein 1 Precursor |
| PTN | NM_002825 | pleiotrophin |
| CDH6 | NM_004932 | cadherin 6, type 2, K-cadherin (fetal kidney) (CDH6) |
| C13orf27 | NM_138779 | chromosome 13 open reading frame 27 |
| PRDX2 | NM_005809 | peroxiredoxin 2 |
| DCBLD1 | NM_173674 | discoidin, CUB and LCCL domain containing 1 |
| KIF5C | NM_004522 | kinesin family member 5C |
| IAH1 | NM_001039613 | isoamyl acetate-hydrolyzing esterase 1 homolog |
| PDGFRL | NM_006207 | rplatelet-derived growth factor receptor-like |
| NKAIN1 | NM_024522 | Na+/K+ transporting ATPase interacting 1 |
| AGPAT9 | NM_032717 | 1-acylglycerol-3-phosphate O-acyltransferase 9 |
| SPA17 | NM_017425 | sperm autoantigenic protein 17 |
| ENPP4 | NM_014936 | ectonucleotide pyrophosphatase/phosphodiesterase 4 |
| MKX | NM_173576 | mohawk homeobox |
| TMEM17 | NM_198276 | transmembrane protein 17 |
| MPP1 | NM_002436 | membrane protein, palmitoylated 1 |
| AK024680 | AK024680 | cDNA: FLJ21027 fis, clone CAE07110 |
| GAS7 | NM_201433 | growth arrest-specific 7 |
| C1QTNF5 | NM_015645 | C1q and tumor necrosis factor related protein 5 |
| CD33 | NM_001772 | CD33 molecule |
| SLC19A1 | NM_194255 | solute carrier family 19 (folate transporter), member 1 |
| TOX3 | NM_001146188 | TOX high mobility group box family member 3 |
| ARC | NM_015193 | activity-regulated cytoskeleton-associated protein |
| A2LD1 | NM_033110 | AIG2-like domain 1 |
| ITGB3 | S70348 | integrin beta 3 mRNA |
| EFNB3 | NM_001406 | ephrin-B3 |
| P2RX5 | NM_002561 | purinergic receptor P2X, ligand-gated ion channel, 5 |
| PWWP2B | NM_138499 | PWWP domain containing 2B |
| HOXA9 | NM_152739 | homeobox A9 |
| MXRA5 | NM_015419 | matrix-remodelling associated 5 |
| TNFRSF1B | NM_001066 | tumor necrosis factor receptor superfamily, member 1B |
| MDK | NM_001012334 | midkine (neurite growth-promoting factor 2) |
| CCL2 | NM_002982 | chemokine (C-C motif) ligand 2 |
| HAND2 | NM_021973 | heart and neural crest derivatives expressed 2 |
| C2CD2 | NM_015500 | C2 calcium-dependent domain containing 2 |
| MCTP2 | AL832717 | cDNA DKFZp313B039 |
| KCNK5 | NM_003740 | potassium channel, subfamily K, member 5 |

| RILP | NM_031430 | Rab interacting lysosomal protein |
| --- | --- | --- |
| ALDH1A3 | NM_000693 | aldehyde dehydrogenase 1 family, member A3 |
| HOXD1 | NM_024501 | homeobox D1 |
| SLC2A10 | NM_030777 | solute carrier family 2 (facilitated glucose transporter) |
| ACSS1 | NM_032501 | acyl-CoA synthetase short-chain family member 1 |
| tcag7.1307 | AK125809 | cDNA FLJ43821 fis, clone TESTI4002290 |
| TFF3 | ENST00000291525 | Trefoil factor 3 Precursor (Intestinal trefoil factor) |
| CGNL1 | NM_032866 | cingulin-like 1 |
| EDA | NM_001399 | ectodysplasin A transcript variant 1 |
| NDRG2 | NM_201535 | NDRG family member 2 transcript variant 1 |
| HOXB13 | NM_006361 | homeobox B13 |
| NME4 | NM_005009 | non-metastatic cells 4 |
| PTPN3 | NM_002829 | protein tyrosine phosphatase, non-receptor type 3 |
| SRPX | NM_006307 | sushi-repeat-containing protein, X-linked |
| PTGR1 | NM_012212 | prostaglandin reductase 1 |
| BC028039 | BC028039 | thymosin beta15b |
| BDH1 | NM_203314 | 3-hydroxybutyrate dehydrogenase, type 1 |
| BLVRA | NM_000712 | biliverdin reductase A |
